# Pigmented Odontoma of the Upper Jaw

**Published:** 1915-10

**Authors:** 


**Pigmented Odontoma of the Upper Jaw.** A. V. Moschcowitz, (Annals of Surgery, March, 1915, Arch. of Paed., July). A swelling was first noticed in the right upper jaw of the infant at six weeks of age. This was incised twice and a rudimentary tooth removed. The swelling increased to the size of an English walnut and bled freely at every touch. A section was removed and examined. It proved to be a pigmented odontoma Because of the age of the infant the use of the X-ray was advised but no benefit obtained. Moschcowitz is now considering extirpation of the tumor with the knife or the thermo-cautery. This is probably a malignant tumor. They are of two sorts—hard or crown odontoma, made up of dentine or cement substance, and soft or pulp growths, including periodontium.

